# Pedigree and genotyping quality analyses of over 10,000 DNA samples from the Generation Scotland: Scottish Family Health Study

**DOI:** 10.1186/1471-2350-14-38

**Published:** 2013-03-22

**Authors:** Shona M Kerr, Archie Campbell, Lee Murphy, Caroline Hayward, Cathy Jackson, Louise V Wain, Martin D Tobin, Anna Dominiczak, Andrew Morris, Blair H Smith, David J Porteous

**Affiliations:** 1 Centre for Molecular Medicine, University of Edinburgh, Institute of Genetics and Molecular Medicine, Western General Hospital, Crewe Road, Edinburgh, UK; 2 Wellcome Trust Clinical Research Facility, University of Edinburgh, Western General Hospital, Crewe Road, Edinburgh, UK; 3 School of Medicine, University of St Andrews, St Andrews, UK; 4 Department of Health Sciences, University of Leicester, Leicester, UK; 5 Division of Cardiovascular and Medical Sciences, University of Glasgow, Glasgow, UK; 6 Medical Research Institute, University of Dundee, Dundee, UK

**Keywords:** Genetics, SNP Genotyping, Parent-child trios, Error rate, Non paternity, Generation Scotland, Biobank

## Abstract

**Background:**

Generation Scotland: Scottish Family Health Study (GS:SFHS) is a family-based biobank of 24,000 participants with rich phenotype and DNA available for genetic research. This paper describes the laboratory results from genotyping 32 single nucleotide polymorphisms (SNPs) on DNA from over 10,000 participants who attended GS:SFHS research clinics. The analysis described here was undertaken to test the quality of genetic information available to researchers. The success rate of each marker genotyped (call rate), minor allele frequency and adherence to Mendelian inheritance are presented. The few deviations in marker transmission in the 925 parent-child trios analysed were assessed as to whether they were likely to be miscalled genotypes, data or sample handling errors, or pedigree inaccuracies including non-paternity.

**Methods:**

The first 10,450 GS:SFHS clinic participants who had spirometry and smoking data available and DNA extracted were selected. 32 SNPs were assayed, chosen as part of a replication experiment from a Genome-Wide Association Study meta-analysis of lung function.

**Results:**

In total 325,336 genotypes were returned. The overall project pass rate (32 SNPs on 10,450 samples) was 97.29%. A total of 925 parent-child trios were assessed for transmission of the SNP markers, with 16 trios indicating evidence of inconsistency in the recorded pedigrees.

**Conclusions:**

The Generation Scotland: Scottish Family Health Study used well-validated study methods and can produce good quality genetic data, with a low error rate. The GS:SFHS DNA samples are of high quality and the family groups were recorded and processed with accuracy during collection of the cohort.

## Background

Generation Scotland is a multi-institution, cross-disciplinary collaboration that has created an ethically sound, family- and population-based resource for identifying the genetic basis of common complex diseases [1,2]. The Generation Scotland: Scottish Family Health Study (GS:SFHS) has DNA and socio-demographic and clinical data from ~24,000 volunteers from across Scotland. The ethnicity of the cohort is 99% white, with 96% born in the UK, 87% in Scotland. Specific features of GS:SFHS include the family-based recruitment, with the intent of obtaining family groups; breadth and depth of phenotype information; consent and mechanisms for linkage of all data to comprehensive routine healthcare records; “broad” consent from participants to use their data and samples for a wide range of medical research, and for re-contact. These features were designed to maximise the power of the resource to identify, replicate or control for genetic factors associated with a wide spectrum of illnesses and risk factors, both now and in the future [3]. Potential participants were identified at random from those aged 35–65 years from the lists of collaborating general medical practices, and invited to participate and to identify at least one first-degree relative aged at least 18 years who would also participate [3]. This paper describes analysis of DNA samples from more than 10,000 of the participants in GS:SFHS for genotyping and pedigree quality. This was undertaken to test the quality of genotyping, to inform researchers interested in genetic research using the GS resource.

## Methods

### Extraction and storage of DNA

All samples from Generation Scotland participants were collected, processed and stored using standard operating procedures and managed through a laboratory information management system (LIMS). Blood was taken from consenting participants in GS research clinics using standard venepuncture procedures and collected in a 9 ml EDTA tube. Each blood sample was processed for DNA extraction using a Nucleon Kit (Tepnel Life Science) with the BACC3 protocol. The precipitated DNA was hooked out and placed directly into a labelled 2.0 ml microtube (Scientific Specialities Inc) containing 1 ml TE buffer pH 7.5 (10 mM Tris-Cl pH 7.5, 1 mM EDTA pH 8.0). Postal participants and clinic participants from whom sufficient blood was not obtained were invited to provide a sample of saliva in an Oragene OG-250 saliva kit, for DNA extraction. DNA was extracted from these into similar microtubes by a standard protocol (DNA Genotek). Microtubes were rotated for 2 weeks at room temperature until DNA was fully re-suspended. DNA concentrations (ng/μl) were determined for all samples using the Picogreen method (Invitrogen). Eight out of every batch of 92 samples were electrophoresed on a 1% agarose gel to test for integrity of the DNA and were all satisfactory, and were also run on a NanoDrop (Thermo Scientific) to confirm DNA yield and to determine levels of protein and RNA contamination. 500 μl of each DNA master stock were transferred to a deep well plate then normalised to 50 ng/μl to make working stock plates. The remaining 500 μl were archived in a microtube at −40°C.

### Open array genotyping

DNA from the first 10,450 GS:SFHS clinic participants that had spirometry, smoking data and DNA extracted was analysed, as part of a replication experiment from a Genome-Wide Association Study meta-analysis of lung function [4]. Each plate of DNA held 95 samples, with one well containing only TE buffer as a no template control. The OpenArray genotyping system (Applied Biosystems) uses nanolitre fluidic technology in conjunction with TaqMan® chemistry to enable high-throughput and low cost workflows. Thirty of the SNPs were pre-designed ABI assays and two SNPs were custom designed. After thermal cycling, the OpenArray was imaged on an OpenArray NT scanner as an end point assay and genotypes were called using Genotyper Analysis software v1.0.1. All SNPs were visually examined for any clustering issues. The OpenArray genotype data was imported into a MySQL v5.1 database and analysed using SQL scripts. The results were verified with Pedstats software within the MERLIN package [5].

### Ethical issues

All components of GS:SFHS have received ethical approval from the NHS Tayside Committee on Medical Research Ethics (REC Reference Number: 05/S1401/89). GS:SFHS has been granted Research Tissue Bank status by the Tayside Committee on Medical Research Ethics (REC Reference Number: 10/S1402/20) providing generic ethical approval for a wide range of uses within medical research.

## Results

### Sample extraction

The GS:SFHS DNA samples analysed all passed the routine quality control tests of intactness and purity described in Methods. Yield of DNA was high, with a low rate of extraction failure. DNA was obtained from over 98% of the 23,960 GS:SFHS participants. Of these, 2.5% had a total yield of less than 30 μg of genomic DNA, while the yield of the other 97.5% had an mean of 272 μg and a median of 232 μg. If necessary, at some future point DNA stocks of low yield samples could be replenished through the technique of whole genome amplification [6], or by re-contact and re-sampling of participants.

### SNP genotyping

Thirty-two SNP markers were chosen as part of a follow-up replication experiment from a Genome-Wide Association Study meta-analysis on lung function, which has provided insights into genetic causes of chronic pulmonary disease [4]. The details of the SNPs genotyped are summarised in Table 1. They lie on eleven different chromosomes and have minor allele frequencies ranging from 4.3% to 49.6%, in good or excellent agreement with rates recorded in populations of European ancestry in the dbSNP database [7]. There are 27 independent SNPs, as indicated by superscripts a-d showing the four separate groups of related markers in Table 1.

**Table 1:** Summary of SNP markers analysed

**dbSNP ID**	**Chr.**	**Major allele**	**Minor allele**	**Minor allele frequency**	**Call rate**	**Method**
rs2284746	1	C	G	47.7%	91.6%	OpenArray
rs993925	1	C	T	34.9%	97.6%	OpenArray
rs12477314	2	C	T	20.2%	97.7%	OpenArray
rs2544527	2	C	T	35.9%	97.8%	OpenArray
rs1344555	3	C	T	21.3%	98.0%	OpenArray
rs1529672	3	C	A	17.8%	98.5%	TaqMan
rs9310995	3	T	C	43.6%	98.2%	OpenArray
rs1541374	4	G	T	34.6%	94.1%	OpenArray
rs10067603	5	A	G	24.3%	96.2%	OpenArray
rs1551943	5	G	A	23.6%	97.3%	OpenArray
rs153916	5	T	C	45.8%	97.9%	OpenArray
rs2798641	6	C	T	17.6%	98.3%	OpenArray
rs1928168	6	C	T	49.6%	98.3%	OpenArray
rs2855812^a^	6	G	T	26.4%	97.1%	OpenArray
rs2857595^a^	6	G	A	21.5%	95.4%	OpenArray
rs3094548	6	C	G	35.2%	97.5%	OpenArray
rs3734729	6	A	G	4.3%	97.9%	OpenArray
rs6903823	6	A	G	26.8%	99.4%	TaqMan
rs11001819	10	A	G	48.9%	97.1%	OpenArray
rs7068966^b^	10	T	C	48.5%	98.1%	OpenArray
rs1878798^b^	10	G	C	46.2%	96.9%	OpenArray
rs1036429	12	C	T	21.3%	98.0%	OpenArray
rs11172113	12	T	C	41.3%	97.6%	OpenArray
rs4762767	12	G	A	27.5%	97.7%	OpenArray
rs12914385^c^	15	C	T	38.6%	97.0%	OpenArray
rs2036527^c^	15	G	A	33.8%	97.8%	OpenArray
rs8040868^c^	15	T	C	38.9%	96.3%	OpenArray
rs2865531	16	A	T	40.9%	99.5%	TaqMan
rs12447804^d^	16	C	T	23.4%	95.2%	OpenArray
rs3743563^d^	16	C	T	23.1%	98.6%	OpenArray
rs12716852	16	A	G	45.2%	97.5%	OpenArray
rs9978142	21	A	T	14.2%	97.6%	OpenArray

Legend: The table shows the reference SNP (rs) ID number and chromosome location of all markers, minor allele frequency and call rate (number of successful genotype calls as a percentage of the total samples assayed). The four groups of non-independent SNPs (defined as pair wise linkage disequilibrium r^2^>0.29) are indicated by superscripts a-d.

29 SNPs out of the 32 SNPs chosen gave analysable OpenArray data. The three SNPs that failed on the OpenArray could not be called because individual clusters could not be identified. These SNPs were successfully re-run as Taqman assays on the 7900HT platform (Table 1). Call rates ranged from 91.6% to 99.5% of DNA samples assayed (Table 1). Within the 10,450 DNA samples analysed by OpenArray, there were 289 samples derived from saliva, with an average call rate of 96.22%. There were 10,161 samples derived from blood and these had a slightly higher call rate of 97.12%.

### Use of family data to check study processes - analysis of marker transmission in complete trios

There may be occasional sample mix ups in any research clinic and laboratory, but careful adherence to standard operating procedures and following good clinical practice and good laboratory practice should minimise such errors close to zero. However, the inclusion of family structures allows some independent verification of how well the study has been conducted, as the laboratory performing the genotyping is blinded to the family structure.

**Figure 1:**
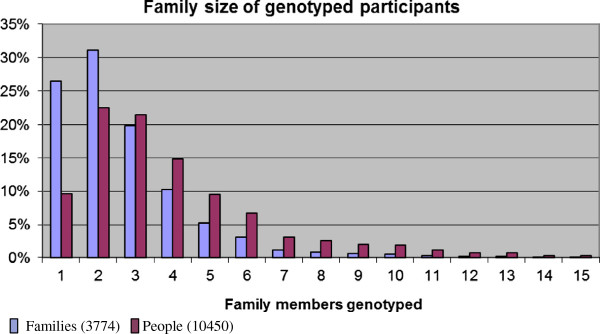
Family size of genotyped participants.

Within the samples analysed, there are many different and sometimes complex family structures of up to three generations and with extensive kinship, both near and distant. The 10,450 GS:SFHS participants genotyped can be grouped in 3,774 families. The distribution of family size is shown in Figure 1. The family structure of the sample set assayed is representative of that in the whole GS:SFHS cohort of ~24,000 people. The largest family group in this genotyped subset of the SFHS cohort has 24 members, within a complex pedigree. For the purposes of this analysis, the focus was on complete trios (two parents and a child). The participants genotyped contained 925 such trios which were not all independent, instead spread across 576 families with up to six children (Table 2).

**Table 2:** Number of complete trios genotyped within families of two parents and one or more offspring

**Number of children**	**Number of families**	**Parent-child trios**
1	288	288
2	237	474
3	44	132
4	5	20
5	1	5
6	1	6
**Total**	**576**	**925**

Legend: The number of children, families and parent-child trios genotyped is shown.

**Figure 2:**
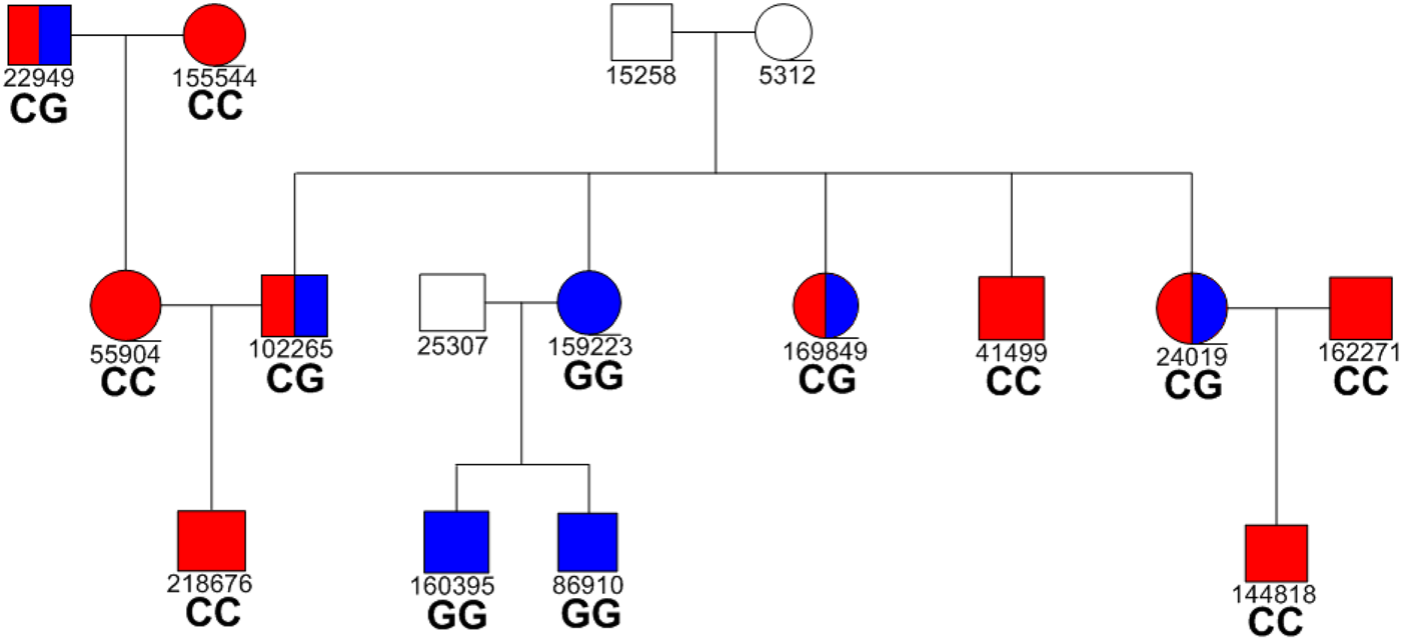
**Coloured symbols represent family members who were genotyped, open symbol represents people not recruited into GS: SFHS.** Unique participants IDs allocated for this project are shown under each symbol. Genotyping results for the SNP rs3094548 in family F289 are shown, with blue reprenting the G allele and red the C allele.

One of the largest families, ID number F289, contained 16 members in three generations, 13 of whom were genotyped in this study (Figure 2). The three people not genotyped are represented by un-shaded symbols and were not recruited into GS:SFHS, but their existence is required to draw the pedigree. There are three complete trios within this single pedigree. The genotyping data for one of the markers that is informative for this family, rs3094548, are shown. All trios in this family show inheritance patterns consistent with Mendelian laws for this and the other 31 markers tested. A possible total of up to 29,600 genotype results (925 × 32) would theoretically be available for analysis. After removing undetermined sample calls, 27,471 results were obtained, of which the overall parentage statistics show 27,282 (99.31%) to be completely consistent with the recorded pedigrees. The results of the 189 analyses inconsistent with pedigree, affecting 129 trios, are detailed in Table 3.

**Table 3:** Summary of SNP data inconsistent with pedigree

**Number**	**Trios**	**Trios excluding rs2284746 data**	**Trios excluding rs2284746 and excluding 5 non-independent SNPs**
**9**	1	1	0
**8**	2	2	2
**7**	1	1	1
**6**	0	0	1
**4**	2	2	2
**3**	4	4	4
**2**	18	14	6
**1**	101	73	33
**total**	**129**	**97**	**49**

Legend: The number of inconsistent SNPs is given, for three categories of marker sets.

Genotype error is a likely explanation for many of the instances of lack of adherence to expected inheritance, as 101 of the 129 trios are only inconsistent for one SNP out of the 32 tested (Table 3). The SNP with the lowest genotype call rate out of all 32 is rs2284746 (Table 1). Excluding the genotype data from this SNP reduces the total number of inconsistent trios to 97, while the 10 most inconsistent trios are unchanged (Table 3). This analysis was repeated after excluding five non-independent SNPs, indicated by the superscripts in Table 1. In each of the four such sets of SNPs, the marker with the highest genotyping call rate was retained for analysis, leaving a total of 27 SNPs, from which rs2284746 was again removed. This reduced the total number of trios with inconsistencies to 49, of which 16 had two or more markers which were not consistent with the recorded pedigree.

## Discussion

An access process has been defined for Generation Scotland resources and is fully operational. Family-based designs for genome-wide association studies are of renewed interest [8]. High density genome-wide genotype data on 10,000 GS:SFHS DNA samples will soon be generated, together with whole exome sequencing of DNA from nearly 1,000 participants. However, it is important that before resources are committed to such large scale genotyping or DNA sequencing, family relationships are verified where possible and quality of the samples is confirmed. Confident identification of pedigree errors could also allow correction of the stored data, thus improving its accuracy.

This study found high or very high call rates for DNA from all of the samples tested, and for all of the 32 SNPs assayed, and a high rate of consistency with recorded pedigrees. The 32 SNPs in this analysis were chosen for a lung function study [4], rather than for pedigree testing, but provide a good range of allele frequencies in this Northern European population (Table 1), and most are independent, allowing testing of the recorded family structures. The relative frequency of the major and minor alleles is an important determinant of how informative a biallelic SNP assay is in a pedigree. Error detection rates are lowest when the two alleles have equal frequencies [9], i.e. there is a higher chance that any (unrelated) trio would show a genotype consistent with Mendelian inheritance, despite not actually being related. Conversely, with a minor allele frequency of 10%, it is more likely that an inheritance discrepancy would be evident in an incorrect pedigree [10]. Detection rates are generally lower when the error occurs in a parent than in an offspring [9]. Teo *et al*[11] described *Nucl3ar* software, which assesses the extent of pedigree inconsistent genotype configurations in the presence of genotyping errors. This recognised the problem which was addressed here by analysis using other software as described.

Any pedigree errors will be unequivocally apparent with higher throughput data such as GWAS, but the expected inconsistency rate when a pedigree error is present with current data would require detailed simulation to calculate, as it depends on allele frequency in the population, and in the families studied. There are 27 possible genotype configurations for genotype data at a SNP for the three individuals in a trio, of which 15 are pedigree consistent and 12 are pedigree inconsistent (see Teo *et al*[11] Figure 2 for a summary diagram). The few inconsistent trios detected here could have arisen because of errors in pedigree data collection, sample handling or labelling errors in the clinic or lab, in the sample selection for genotyping, or genotyping call errors. Pedigree data was recorded during the volunteer recruitment process, and has not been independently verified. Cross-checking with the General Register Office for Scotland would be laborious and outside the terms of consent. Participants could also have failed to disclose adoption, or there could be a different biological father to that recorded.

Reliable estimates for non-paternity rates are difficult to establish, with high quoted rates often proving to be anecdotal [12]. A median rate of paternal discrepancy of 3.7% was reported in a review of 17 populations, studied for reasons other than disputed paternity [13]. The true rate may lie closer to 1% in the UK and elsewhere in Europe [14,15]. The analysis of 925 trios presented here (Table 3) is unable to unequivocally distinguish the source of all of the few apparent errors present, with inconsistencies in the father, mother or either parent apparently occurring at approximately equal frequency. Consideration of the 16 trios with two or more independent SNPs showing inconsistency (Table 3, fourth column) shows that in 3 of the trios the inconsistency in the child is not with the genotype of the mother, indicating a maximum estimated non-paternity rate in the Scottish Family Health Study of less than 1.5% (13 trios out of 925 analysed). The true rate is likely to be considerably lower, as it is unlikely that all discrepant results are caused by incorrect pedigrees. These relatively low rates may in part be due to non-participation in the study by women who knew the paternity of their child was uncertain. Participants were informed (in the information online) that “As part of the Scottish Family Health Study, researchers will perform tests to check that family members are genetically related, because this is essential for the success of the study. The researchers who carry out these tests will not know, or be able to find out, the identities of the people who gave the samples. Generation Scotland will not pass the results of family testing back to families”. Our study provides a first estimate of these kinds of errors in the Scottish Family Health Study. More refined estimates will be generated once it is feasible to run genome-wide genotyping arrays for these samples, as the extensive information on such arrays will improve both the sensitivity of error detection and the resolution of genotyping errors from pedigree inconsistencies. Whilst genome-wide genotyping lies outside the scope of the current study, the wealth of phenotype data available in GS:SFHS mean that it will prove a rich resource for genome-wide association studies in the near future.

## Conclusions

The analyses presented here provide evidence that the GS:SFHS DNA samples and recorded pedigrees are of high quality and suitable for genetic analyses. The systems for collection of family structure data and linkage of data and samples are fit for purpose.

## Competing interests

The authors declare that they have no competing interest.

## Authors’ contributions

All authors contributed to the writing of the manuscript, in an iterative manner. SK was the project manager. LM managed the laboratory work to extract and characterise DNA and genotype it. AC managed the genotype database and performed the required data analyses, with input from CH. AM, DP, BS and AD are Principal Investigators for GS:SFHS. MT, CJ and LW led the analyses for the original purpose of studying lung function. The main text was drafted by SK, with comments and amendments made by all authors, who have each read and approved the final manuscript.

## Pre-publication history

The pre-publication history for this paper can be accessed here:

http://www.biomedcentral.com/1471-2350/14/38/prepub
